# Saudi Women and Vision 2030: Bridging the Gap?

**DOI:** 10.3390/bs11100132

**Published:** 2021-09-27

**Authors:** Wafaa Saleh, Areej Malibari

**Affiliations:** 1 College of Engineering, Princess Nourah Bint Abdulrahman University, Riyadh 84428, Saudi Arabia; 2 School of Engineering and the Built Environment, Edinburgh Napier University, Edinburgh EH10 5D, UK; 3 Department of Computer Science, Faculty of Computing and Information Technology, King Abdulaziz University, Jeddah 21488, Saudi Arabia; aamalibari1@kau.edu.sa

**Keywords:** Saudi women travel behaviour, private car driving, women driving

## Abstract

(1) Background, Travel characteristics of Saudi women contrast significantly from those in the west. This is not only because they have different culture, attitudes and preferences but also until recently, Saudi women were not allowed to drive. In 2018, they were granted the right to drive. It has been anticipated that enabling women to drive will improve their mobility and employability. (2) Methods: This study presents a qualitative study into factors affecting Saudi women’s travel decisions “before” and “after” enabling women to drive in the Kingdom. Two six “before” and “after” focus groups have been carried out to investigate the decision-making process associated with Saudi women’s travel, available options of travel and perception of Saudi women towards private car driving. (3) Results: The results reveal that main travelling options for professional and high-income women is a private driver in the “before” scenario and a ride-share option with a family member. In the “after” scenario, high income professional women prefer “drive own car” option. Moreover, many of the participants indicated that it is likely that they might keep private drivers as well. (4) Conclusion. The results from this research indicate that there has been significant change in travel characteristics, attitudes and behaviour of Saudi women since they were granted the right to drive. This is likely to have significant implications for decision and policy makers. Further research into potential impacts of the current situation on car ownership and use, impacts on public transport system, environmental impacts and sustainability is needed.

## 1. Introduction

Until recently, Saudi Arabia was the only country in the world that did not allow women to drive. However, in September 2017, a royal decree granted women the right to drive vehicles, a move which took effect in June 2018. Even though Saudi Arabia was ranked 138 out of 144 countries according to the 2017 World Economic Forum’s Global Gender Gap Report, in 2018, the country was elected as new member to the Executive Board of the United Nations Entity for Gender Equality and the Empowerment of Women. It has been anticipated therefore, that enabling women to drive will improve their mobility and increasing the contribution of women in the labour force from 22% to 30% is a declared objective of the Saudi Arabia 2030 vision (Saudi Vision 2030 is a plan to reduce Saudi Arabia’s dependence on oil, diversify its economy and develop public service sectors such as health, education, infrastructure, recreation and tourism). This will also promote the country’s stand on gender equality programs and achieving Kingdom’s 2030 Vision.

With the growing economy and developments in Saudi Arabia as well as the increasing connections with the western societies, there is an urgent need for greater and richer understanding of the perceptions and attitudes of all members of this society. Factors relating to travel decisions, attitudes and beliefs of Saudi women before and after enabling women to drive are still ambiguous and will take a long time to comprehend and resolve as they are caught up in a larger debate on the role of religion and cultural traditions in their society.

This study presents qualitative research into factors affecting women’s travel decisions and choices in Saudi Arabia “before” and “after” enabling women to drive. The research was conducted using focus group surveys. Two six “before” and “after” focus groups have been carried out to investigate the three main research themes: the decision-making process associated with Saudi women’s travel, options available for travel to Saudi women and factors affecting choices and perception and behaviour of women towards driving own cars.

## 2. Literature Review

### 2.1. Gender Differences in Travel Patterns

A large amount of research has shown that women in general have significantly different travel patterns to men. A variety of factors that may influence gender differences in travel behaviour such as age, household size, education, driving license, car ownership, income, workplace and accessibility [1,2,3,4]. Other factors such as culture and institutions are also relevant and affect travel behaviour and attitudes. The mobility patterns in relation to culture and household structure give important insights into transport patterns and are useful for investigating gender differences [5].

Much research on gender and travel remains focused on linking transportation access with employment opportunities [6,7]. Generally, women commute shorter distances than men [6], but women engage in highly complex travel and time use patterns due to their increased role in the workplace, increased car use and still-high share of child caretaking and domestic responsibilities [7,8]. Similarly, [9] found that married women adjust commuting time and work hours to suit the life cycle of the family. Ref. [10] report that Swedish women have a shorter commute than men regardless of employment sector, education level and family situation [11] found that the effect of home production on commuting time for women is more than double the effect for men and responsibilities such as childcare impose more restrictions on commuting time for women, compared to men.

Women have been recognised in a number of studies as being more likely to adopt sustainable travel behaviours compared with men. In Sweden [12] found that women were more willing to reduce their use of the car than men, more positive towards reducing the environmental impact of travel modes and more positive towards ecological issues.

Recent research in the UK [13] indicates that young women now travel more than young men and that young men travel substantially less today than previous generations of young men. The authors conclude that different generations face different socio-economic contexts which impact on mobility trends. Similarly, studies in Germany [14] have also found that gender differences in car travel are disappearing amongst the younger generation.

Over the years, there has been considerable research into gender differences in travel patterns in developed urban western societies and, more recently, some research in developing countries. In most developing countries, the effect of traditional cultural factors, including religion, gender inequalities, type of employment and accessibility, all have significant impacts on women’s travel behaviour [15,16,17,18,19,20,21]. In a review of gender and transport in less developed countries, ref. [21] found that women are less likely than men to have access to motorised means of transport and be more dependent on either public transport or walking. In addition, it was found that women tend to make more activity trips weekly than men. These include childcare, personal healthcare, shopping, fetching water, waste disposal, social functions and religious purposes, whilst men’s travel activities are mainly for work and school trips as well as recreation.

Compared to western societies, little is known about gender differences in travel patterns in Arab nations and it is important to gain an in-depth understanding of the decision-making process as well as the factors which influence women’s travel decisions. In Arab countries, women’s travel attitudes and behaviour are not only affected by economic and geographical factors but also by other social and cultural differences and restrictions. A small number of studies have attempted to examine gendered differences in travel behaviour in the Arab world [15,22].

In a comparison of commuting times for males and females in Egypt, ref. [23] found that statistically significant differences emerged across virtually all groups of male and females with similar characteristics (e.g., education, marital status, type of work etc.). They also considered geographical mobility in the Egyptian labour market between 1988 and 1998. They concluded that young single males have had to significantly increase their geographical mobility to access regular paid work in the private sector. No such increase in geographical mobility could be detected for female new entrants, resulting in a growing gender gap in geographical mobility rates over the 1990s decade.

Ref. [24] showed that in Libya, demographic variables such as age and gender contributed significantly to explain mode choice behaviour. Furthermore, they found that men are less likely than women to shift to public transport.

Other researchers have observed gender differences in students travel behaviour in Arab countries. Ref. [25] Investigated the travel characteristics of female college students in Saudi Arabia. She found that 57% travelled as car (or van) passengers and 39% travelled by bus and over half (53%) were captives to their current mode of transport.

### 2.2. Saudi Arabia

Saudi Arabia is an Islamic country, where most Saudis practice the Islamic religion and their beliefs are reflected in all aspects of public life [26,27] Littlewood & Yousuf, 2000). Culture and traditions also play important roles in forming norms and beliefs. Recently in Saudi Arabia, car ownership and use have increased over a short period of time due to cheap petrol prices, which has implications on the business and economic development of the country. Moreover, recently women were allowed to drive cars in Saudi Arabia which might have impacts on car ownership and use in the country.

The 2017 World Economic Forum’s Global Gender Gap Report ranked Saudi Arabia amongst the lowest 3 countries (142nd out of 144) in terms of female economic participation and opportunity. However, in terms of educational attainment, Saudi Arabia is ranked higher (96th out of 144). The female economic participation rate stands at 20% (compared to 78% for males). The fact that women were not allowed to drive represented a cause of restriction of mobility and business and economic opportunities. In recent years significant efforts have been made by the government to increase women’s contribution in various development sectors. Ref. [28] presented results showing higher income and education achievements for Saudi women over men in some work sectors, especially in education.

The public transport system is limited in Saudi Arabian’s urban and most people use their own vehicles instead. Buses operate in Saudi Arabia’s intercity and travel to and from neighboring countries. In the past, Saudi women and men were generally discouraged from using public transport, that were mainly used by the expatriates. In 2014, ride-hailing companies Careem and Uber began operating in Saudi Arabia and women then accounted for around four-fifths of passengers with these companies. In the very near future, public transport mode share is likely to increase due to the construction of a metro system in Riyadh which is due to start operation in 2022.

Before women were allowed to drive, the main travel options that were available to women in Saudi Arabia were: passenger in informal car-share (e.g., travelling with family member), passenger in a car share (formally organised), passenger (with private driver chauffeur), passenger (with contracted driver) or taxi. In a typical Saudi household, the most traditional and common travel option for women is a passenger in an informal car share. The husband, older son and/or an uncle, a brother or a grandfather were taking part in these tasks as appropriate. In addition, a private driver, or a number of them, has become a common feature in many Saudi households. More recently, since allowing women to drive in 2018 and with the huge income growth in the country, the women’s access to private cars use has seen a sharp trend.

It is important to investigate the impact of the absence of “drive a car” option had on Saudi women’s perceptions and behaviour towards owning and driving a car. This study presents qualitative research into factors affecting women’s travel decisions and choices in the country “before” and “after” enabling women to drive. Two six “before” and “after” focus groups to investigate three main research themes: the decision making process associated with Saudi women’s travel, options of travel available to them and factors affecting them and perception and behaviour of women towards driving own cars.

## 3. Methodology

### 3.1. Overview

Focus groups were undertaken in order to allow in-depth exploration of the perception and behaviour of Saudi women and their travel characteristics. The impact of the absence of “drive a car” option had on Saudi women’s perceptions and behaviour towards owning and driving a car has also been investigated. In total, two six “before” and “after” focus groups were conducted to investigate research themes. These were: (1) the decision making process associated with Saudi women’s travel, (2) options of travel available to them and (3) factors affecting them and perception and behaviour of women towards driving own cars.

The overall aim of these focus groups was to develop an understanding of the decision-making process for women’s travel choices and the views and preferences of Saudi women about travel options in particular before and after women were allowed to drive.

Focus groups are usually conduced as groups of individuals selected and assembled by researchers to discuss and comment on, from personal experience, the topic that is the subject of the investigation [29]. They are mainly used in qualitative research. They provide a forum for participants to share their attitudes, feelings and beliefs and perhaps reach a consensus on a topic. The literature suggest an ideal focus group size of 6–8 participants plus the moderator. Other arrangements have also been suggested in the literature (see for example [30]. Focus groups provide a descriptive or explanatory rather than a quantitative analogy approach. The sample is typically demonstrative rather than representative of the population, as expected in quantitative methods of data collection and analysis [30,31]. The advantage of this method of data collection is that it allows an in-depth investigation of the topic or phenomena under consideration, including the selection of the sample. The sample is normally chosen according to knowledge or experience of the research topic or by any other socio or economic characteristics.

Whilst a heterogeneous group may provide a wider variety of responses, shared characteristics across the group can encourage all participants to engage in the research discussion and limit unnecessary conflict (see [30,32,33]. In this research, it was decided to form homogeneous focus groups because the shared characteristics of the group members would encourage all participants to engage and also in order to minimise large conflicting opinions within each group. Homogenous groups of women in terms of employability were sought to reduce variations since the topic is controversial.

The number of focus groups required depends on the amount of information needed. More focus groups are needed for more complex questions and fewer groups are needed when the population is homogenous or the question is simple. Even though there are no firm guidelines regarding the number of focus groups, most studies use at least two groups and few studies use more than four groups [34].

### 3.2. Recruitment and Participants

Participants in the focus group surveys were recruited from within shopping malls in Riyadh city in Saudi Arabia in November 2014 for the “before” scenario and in November 2019 for the “after” scenario.

In both surveys, female individuals were approached during evening prayer times where all the shops were closed and all women were either praying in the praying rooms or sitting in the cafes waiting for the shops to open. They were provided with a form (a very brief questionnaire) explaining the main purpose of the focus group surveys and exploring their willingness to take part in one of them. Those women who were willing to participate in the focus group surveys provided their contact details and were assigned to one of the focus groups depending on their characteristics. The focus groups took place at the mall on the days following the day of recruitment.

The sample obtained (30 women) enabled six focus groups to be conducted for each of the “before” and “after” surveys. Each focus group was homogeneous in terms of employment status (i.e., working, non-working or university students) and was made up of five or six women, with the exception of the university students group which had only four members as there were difficulties recruiting more women from this category. A summary of the composition of each group in the before and after surveys are given in Table 1 below.

### 3.3. Procedure

Each group meeting followed a standard format, with the facilitator delivering a brief presentation of the background and consultation, before opening the meeting up to discussion and debate. The purpose of the facilitator was to guide the discussion and ensure that all participants contributed their views. Consistent with standard practice for facilitation focus groups, direct questions were rarely asked and the majority of information came from discussions that were encouraged to develop between focus group participants.

The focus group discussion followed the topic guide summarised below. The topic guide was compiled during discussions with the research team and based on the available literature. This semi-structured format was designed to guide a participant led discussion and reduce interviewer bias. Participants from the group had the opportunity to introduce other topics as long as the discussion remained relevant to the objectives of the research.

The topic guide covered the following:

- Presentation of the background
- Personal introductions, e.g., age, etc.
- General travel opportunities, etc. (including availability of private driver in the “before” survey and availability of car driving option in the “after” survey)
- Attitudes and preferences
- Male members of the household’ role change, if applicable

In each survey, the six focus groups were facilitated by a female moderator whose responsibility was to facilitate the group discussions, make recordings and notes and make sure that every member of the group was engaged. Incentives are normally offered to participants; in this case, a coffee and a cake were offered to each of the respondents over the group meeting. The focus groups were very dynamic and the participants were very enthusiastic. This is despite the fact that they all reported that this type of survey is very new to them, and in fact they have never been surveyed about public or policy issues before.

### 3.4. Thematic Analysis

Thematic analysis was conducted to identify, analyse and report themes within the data. This form of analysis was selected for its suitability in identifying and describing the opinions gained through group discussion. The process followed 5 phases of thematic analysis based on [35]:

- Phase 1: Data familiarisation: meeting notes were read through carefully to gain familiarity with the data content and develop initial ideas.
- Phase 2: Initial coding: Interesting features in the data are grouped into codes. No pre-existing coding frame was required as this method is ‘data driven’
- Phase 3: Searching for themes: sorting the coded data into potential themes and sub-themes
- Phase 4: Reviewing themes: Initial themes are reassessed for their relevance and potential for inclusion
- Phase 5: Reporting

## 4. Discussion

The discussion of the outcomes from the focus groups are presented in this section under the three themes of the research: (1) Section 4.1. Examining the decision-making process related to Saudi women’s travel and the role of men in these decisions; (2) Section 4.2. Available options of travel to Saudi women’s and factors affecting decsions, and (3) Section 4.3. Saudi women’s perceptions and behaviour towards private car driving. In each of these three theams the discussions were summarised for each of the “before” and “after” scenarios.

### 4.1. Examining the Decision-Making Process Related to Saudi Women’s Travel and the Role of Men in These Decisions

#### 4.1.1. Before Scenarios

When the focus group participants were asked about who makes the decisions on issues related to women’s travel, it was very clear that there was a major role for the male head of the family on all decisions related to women’s travel in Saudi Arabia. Over 90% of all participants reported that it was the male member of the family who has to make all arrangements that are to do with private drivers, contracted drivers, employability, registration of vehicles, etc. Over 50% of participants however, reported that some decisions would have to be taken jointly such as who to drive the kids to schools, who to drive the female members of the family, etc. This is because it would usually be the mother who knows all the family members’ travel requirements. One older participant of high income reported that “…all travel matters that relate to the kids are my duty to sort. I have to decide who drives who to places etc. My husband would interfere if there is a problem with the driver, otherwise all my responsibility”. The male member of the family would contribute to other members’ travelling requirements however, where possible and if needed.

The level of intervention seemed to vary amongst the participants with some stating that it depended on issues such as availability and number of private drivers at the family as well as household structure. All participants from larger families supported this account. Other factors that were mentioned include socio-economic characteristics, level of education and income. The reasons discussed for such interventions were either religious, cultural or social. Safety and security of women with private drivers were also stated as reasons for concern in Saudi Arabia since most drivers are foreign. Other male travel-related duties include having the main role in employing a private driver, arranging for a contracted driver and giving permission to travel to the younger females in the family.

The second focus group (non-working women) in the “before” case, stated that their own travel decisions are made with consideration to other family members and conditions and the driver’s availability. Since most of travel needs of this category of women are flexible (no work commitment), travel decisions are always flexible and considerate to others in the family. The male member of the family usually arranges the private driver as discussed or a contracted driver if needed.

Interestingly, the perception of most participants in the focus groups appeared to be supportive of the male role in travel decisions related to them, and saw this as the accepted social norm of the society, religion and the culture. Generally, over 80% of the older participants and about 65% of all participants seemed to be more accepting of this role in the “before scenario”.

A number of participants in the third focus group (university student) indicated that travel choices are very limited as they live on campus, where there are some limited shuttle rides at certain times. Other and more general travel arrangements have to be approved by the woman’s guardian, so their travel activities outside the campus are very restricted. Most recreational activities for this group were taken within the all-female university premises. Other participants of this group, who live at home, reported that they have to communicate/negotiate their travel needs with the family, but in essence travel decisions are their own and it is only the logistics that are sometimes arranged by a male member of the family.

#### 4.1.2. After Scenario

Regarding the role of the male member of the family with travel decisions of women, the perception of most participants in the focus groups appeared to be indicating that role. They were supportive however of the male role in their travel decisions, and saw this as the accepted social norm of the society. Generally, the older women seemed to be more accepting of this role while almost 50% of the younger participants were keen to reflect on alternative possibilities. One older participant said, “they know better, safer to consult with them…”. It was also strongly recognised by all participants that the male dominance in the structure and routine of the decision-making process does marginalise the vulnerable women in the Saudi society. These include older women who have no available male member in the family, divorcees, widowed or lower income women.

In the “after” survey of working women (focus group 4), most women reported that the decisions related to them driving a car would be a jointly agreed with the male head of the family. Similarly, travel arrangements for other members of the family including daughters, young sons (who don’t yet have access to driving a car) or older members of the family, are jointly decided upon.

The younger members of the focus group (non-working women) in the “after” survey, stated that since they have started driving, their travel to many social and recreational activities has become much easier and/or possible. For the older members and lower income categories in this group, they reported that their travel decisions are still made with consideration to other family members and conditions and the driver’s availability. Only one member of students in this group reported that she was in the process of training to acquire a driving license as soon as she will be allowed.

Many of participants in this group reported that “I would try to get a car as soon as I can be able to and can afford”. In that category also, a number of participants from large families and high income reported that the male head member of the house is the one responsible for arranging all travel requirements/logistics/assignments of modes.

In conclusion, in all groups, it appears that the male member of the family does have a role to play in all travel arrangements associated with the women and other family members. This is most likely because of the need to deal with contracting a private driver or a contracted driver. It is also because of the need to carryout routine maintenance, registrations, repairing and other tasks associated with the private cars owned by the family. In Saudi Arabia families are typically of greater number of persons (ten members or more) than of western families and own more than one car in the household. Therefore, it is the male, or the private driver task to sort out all tasks related to private cars in the family. Regarding travel decisions for the family’s members it seems that these are mostly joint decisions nonetheless in most cases there is a male presence in such decisions.

### 4.2. Available Options of Travel to Saudi Women’s and Factors Affecting Decsions

#### 4.2.1. Before Scenarios

When considering the outcomes by focus group segments, working women (focus group 1) reported that they employ a private driver and most of their local travel is carried out using this option. Alternatively, they are the most frequent users of the family’s one private driver. They might offer their own driver to transport other members of the family before and/or after their own journeys. A few participants, with lower income or who work far from their place of residence, reported that they share a ride with other female traveller with a contracted driver.

When they were asked about the main factors for the choice of mode of travel (the private driver), 50% of participants mentioned that the reasons for preferring this option include reliability, safety, security and convenience. For long distance travel, a private driver or a shared ride would be the option; a car sharing with a male member of the family.

The second 50% of the participants agreed that cost is the main factor to favoring the private driver in the survey. “Once you employ a private driver, it will be more cost effective to use him”, one participant stated. “Even though you have to accommodate, employ and pay for the driver’s living expenses, it works out better for the family as there are many individual and family trips during the day which keeps the driver occupied most of the time”, another participant has reported. Several participants in this group pointed out the added values of having a private driver. These include that a private driver would quickly become acquainted with most of the destinations related to the family travel, he will be familiar with family members, can take care of baggage or gear in the car if needed, carryout other tasks as well as the ability to leave child-related luggage and toys in the car. In cases where there are no possibilities to accommodate a private driver, therefore a private driver is not available. Contracted drivers are used in these cases in both scenarios. Moreover, it was stated during the focus groups that “contracted drivers and taxis are expensive, unreliable and unregulated”, one participant detailed.

Non-working women (focus group 2) stated in the “before” survey that they mostly travel as “a passenger with a male member of the family or a friend”, or using a taxi (in most cases this would be a regular taxi driver who has been previously used). This mode was most favoured also by those on a medium or low income in other focus groups.

A number of participants from relatively large families and high income in the “before” survey reported that there is more than one private driver at the household and more than three cars. At times however, all cars and drivers are engaged in driving members of the family. The male head member of the house is the one responsible for arranging all travel requirements/logistics/assignments of modes. A few participants in the sixth group (university students) indicated that they rely on university shuttle transport services most of the time since these are the only available options. Other travel options have to be approved by the family. When at home, university students use the family private driver or acquire a ride with a male member of the family, depending on the trip. Alternatively, they would acquire a contracted driver, but this is an expensive option.

It was also reported that where there was a private driver available, all driving tasks, especially those related to the women in the family, will be the duty of the private driver. An older high-income participant confessed “a private driver is a gem, he does far more than just driving us around, but he has to be trustworthy…”. Where there was no private drivers available at the household, a contracted driver was highly likely to be hired. This driver would be contracted on a regular basis to drive members of the family, especially the children and women, to their required destinations. The advantage of this option is that the driver does not have to be accommodated at the family’s house. A lower income participant reported “I don’t have to accommodate him and be responsible of him …”. However, the disadvantage of this option is that it could well cost more and would be less reliable. A younger participant said “I will buy a car and drive as soon as I can afford it, it would save me loads of money, and be more reliable”. A taxi would be used where there is no available contracted driver, private driver or informal car sharing. However, most people would tend to use a taxi driver who has been used previously by the family and is trusted by them for safety and security reasons.

Women’s age and income are also factors in decisions related to travel. The participants discussed the females age and level of authority over travel decisions. They suggested that older women would have more authority, but would be more conservative.

#### 4.2.2. After Scenario

For the high income working focus group survey (focus group 4), most women reported that they now have access to drive a private car. They reported that this option is most reliable, safe and also prestigious. Those who don’t have access to a private car reported that they employ a private driver or a contracted driver who they use all the time. They saw that employing a private driver is a second best option that provides reliability, safety, security and convenience. Moreover, those who don’t have access to a private car yet, reported that they are in the process of acquiring one.

Of the 15 participants who took part in the “after survey”, 60% reported that they have private car available for them to drive (nine participants). About 88% of those reported that they will keep their private drivers because they will not be able to cope with all driving tasks for the whole family, especially those related to shopping and driving children to schools. It is therefore very likely that after women started driving, there will be an increase in car ownership and use. When participants were probed on this, one working participant of high income said “…this is an important matter, I will definitely have to keep my private driver in order to drive the kids to school, do shopping etc…”. Other-wise, a contracted driver would be the alternative option; to be contracted on regular basis to do schools runs, shopping trips, etc. Few participants also reported that in this case, there would be the need of having an adult present with the children, or an older sibling for safety and security of the younger children. In most cases, the participants reported that they tend to use a taxi driver or a contracted driver who has been employed previously by the family and is trusted by them.

Most participants in the “after survey” agreed that cost is the main factor to favoring the private driver. The higher income members in this group reported that they drive private cars, otherwise they use contracted driver or a taxi. Members who are a medium or low income in this focus group including older members of the family, reported that they mainly use a contracted drivers for most of their travel.

In the same category in the “after” survey, a number of participants from large families and high income reported that three or more female members of the family have had access to a private car since this has become an option. They also reported that they still employ one or more private drivers at the household and own more than five cars in some cases. This reflects the number of members in the household and the level of income as well. At times, all cars and drivers are being engaged in driving members of the family. A number of participants in the sixth group (university students) also indicated that they as soon as they can pass their driving tests, they will be on the road driving their own cars.

In conclusion, in this section, the options available for women travelling include order of preference and affordability, a private driver who will be carrying out many tasks and well trusted by the family. Should a private driver becomes not feasible, then a contracted driver, a shared ride or a taxi were the most commonly used options for travel in the “before” scenarios. In the “after” scenario, the private car seems to be the most attractive option. This option seems to be most preferred especially for high income and working women. In addition, the private car was most preferred by the younger women than the older ones. Lower income participants tend to use a contracted driver of a shared ride.

### 4.3. Saudi Women’s Perceptions and Behaviour towards Private Car Driving

#### 4.3.1. Before Scenarios

In the “before” scenario women where asked about their views on driving a car. About 30% of the higher income group working women reported that they possess a driving license and they do drive when they are abroad. Younger women reported their support, anticipation and backing for allowing women to drive. A younger participant declared “I will buy a car and drive as soon as it becomes legal”. Driving would save me time, money and hassle. Women’s age and income seemed relevant factors that affect women preferences and views on allowing women to drive. While most of middle-aged women were not keen to become involved in these discussions, 80% of the older women were more conservative and saw it as a matter for the society to decide upon.

Most participants who stated that they have started driving, reported that the private car would be paid for by the man, or by the woman herself, then would be principally at her own command. Working through all required tasks that involved acquiring the appropriate training, obtaining driving license and purchasing a car, if there is no available car within the household that can be used by the newly female driver, represent all new experiences that are novel and exciting.

#### 4.3.2. After Scenario

In the “after” survey, the perception of most participants in the focus groups appeared to be very supportive of the idea of enabling women to drive, even for those who weren’t actually driving. Most of the working women of the family have reported that they have started driving and got their own private cars. Of those participants who were working and have access to private cars and started already driving one reported “driving is life…”, another said “driving is freedom”, a third stated “I can get up early and drive to the sea, without having to wait to be driven or be relying on anyone,…”. Women who started driving reported that their male members of the family have been supportive and were more accepting for them to drive. However, over 90% of them reported that all tasks associated with buying the car, sorting out paper work, maintenance of the cars or hiring a driver would still be the duties of the male members of the family. It was also soundly recognised by all participants that since the new regulations are now in place to allow women to drive, the male acceptance of the matter has become much easier. All the participants were appreciating these new regulations. One participant reported “my husband is happy, he feels he is not a driver for the whole family anymore!”.

The perception of most participants in the focus groups appeared to be very supportive of the idea of enabling women to drive in the kingdom. It was seen as an anticipated dream that has come true. Even the older and lower income participants who were unlikely to consider driving as an option, they were in support of the plan. They were in support for the plan for the sake of their community and other women in the family or the community.

## 5. Conclusions, Policy Implications and Direction for Future Research Agenda

### 5.1. Conclusions

There is very limited research about Saudi women, who were until very recently not allowed to drive travel behaviour. This has changed and currently there are hundreds of thousand Saudi women who possess driving licenses and drive. This research is attempting to provide some understanding of the factors affecting Saudi women’s travel decisions, behaviour and choices of modes of travel in the “before” and “after” scenarios. The study has been organised using two sets of six focus group surveys for the “before” and “after” women were allowed to drive in Saudi Arabia. For each of the two “before” and “after” surveys, three groups in each survey, were constructed to investigate differences in perspectives between group participants based on employability, availability of a private driver, household structure, age and income. It should be mentioned here that the age of each group/participant was not identified. This was because it would be inappropriate to ask for an exact age and therefore age groups were identified based on the observer assessment. It would be interesting in future research to acquire more information on more exact age ranges.

Three main research questions have been investigated: (1) the decision-making process associated with Saudi women’s travel options; (2) alternatives available for travel for Saudi women and factors affecting the choices, and perceptions and attitudes towards women driving.

When the focus group participants were asked about who makes the decisions on issues related to women’s travel, it was very clear that there was a major role for the male head of the family on all decisions related to women’s travel in Saudi Arabia. Majority of participants reported that it was the male member of the family who has to make all arrangements that are to do with private drivers, contracted drivers, employability, registration of vehicles, etc. Other participants however, reported that some travel decisions are taken jointly such as who to drive the kids to schools, who to drive the female members of the family, etc.

The level of intervention with women’s travel decisions vary depending on availability and number of private drivers at the family, household structure, socio-economic characteristics, level of education and income of the women. The reasons discussed for such interventions were either religious, cultural or social. Safety and security of women with private drivers were also stated as reasons for concern in Saudi Arabia since most drivers are foreign.

The results from this study reveal that for the employed women, especially those who are in professional jobs, as well high income non-working women, an employed private driver was the main option for travel during the “before” scenario. A contracted driver or a taxi would also be used for women who do not have the privilege of having a private river. Factors associated with choice of mode of travel include cost, safety, reliability and the availability of own car. Other factors include the health conditions of the male members of the family, the number of female members of the family, and the availability nearby of other members of the family who employ private drivers can also affect travel decisions related to women. In the “after” survey, the opinion of most participants in the focus groups appeared to be very supportive of allowing women to drive, even for those who weren’t actually driving. Most of the working women of the family especially those who are of high incomes have reported that they have started driving already their own private cars. Interestingly however, most of them also reported that they are very likely to keep their private drivers for the purpose of driving other members of the family and do other tasks for the household. This might have negative implications on car ownership, congestion and environmental impacts.

### 5.2. Policy Implications

The study results showed that Saudi women are welcoming the new actions of allowing them to drive. This might have implications on vehicle ownership growth in the country and other transport policies. Most women who have started driving are of the high-income population and indicated that they are very likely to keep their own private drivers for the purpose of driving other family members and other household tasks. This is something yet to be verified especially in the aftermath of COVID-19 and the anticipated impacts on the foreign labour force in the country. In addition, the country is in the process of its nationalisation plan. In September, 2020 the Kingdom issued decisions to nationalise some jobs and activities and to enforce the Saudisation policies; the impacts of which are all still to be established.

Vehicle costs and operational costs are currently low in Saudi Arabia as a result of the cheap petrol prices, the high income and low taxes in the country. Once women start buying cars and driving, car ownership might well increase further, in addition to keeping their private drivers. The situation is likely to change overtime, however the right transport policies need to be implemented to maintain a sustainable level of car ownership and use. These policies could include introduction of variable taxes on cars to reflect number of cars per household, engine size, level of emissions, etc.

The rapid increase in vehicle ownership and use will no doubt have significant policy implications for the Kingdom. It will lead to an increase in vehicle emissions and fuel consumption which means acute impacts on the environmental and sustainable programs that the government is currently adopting as part of the Vision 2030 for decent air quality and climate change. The current national policy on the environment will have to be reviewed to recognise these impacts.

The rapid increase in vehicle ownership and use will also represent a threat to the public transportation systems such as Riyadh Metro which represent a huge investment and a state-of-the-art public transportation scheme. The increase could reduce the opportunity for full utilisation on public transportation investments. Policymakers should implement adequate transport policies that would guarantee strong public acceptability and use of these public transportation systems. Additionally, policies that should facilitate the last stages of the journeys (access and egress journeys) are important elements of the public transportation journeys and could influence their attractiveness and use.

Traditionally, the Saudi population were discouraged from using public transportation. Now with women started driving that is coinciding with the operation of the Riyadh metro which represent a huge investment, there is a need to raise awareness and encourage women and men to use the metro system and in general to encourage the use of public transportation systems.

### 5.3. Direction for Future Research Agenda

There is an urgent need for future research in this area in several directions in order enhance understanding, support and improvement of the transport system in Saudi Arabia. Few suggestions are listed below:

1. Investigate and enhance public acceptability for using public transportation systems and reduce car use,
2. Assess possible transport policies that aim at reducing car use and using alternative options.
3. Assessment of the impact of women driving on car ownership and use on the performance of the transportation systems in Saudi cities.
4. Investigation of technological solutions that support reducing vehicle emissions and fuel consumptions.
5. Public transportation fare structure and further measures and policies needed to encourage the use of public transportation system such facilitating the access/egress journeys from home to the stations and vice versa.
6. Impacts of driving various car types and engine capacities on emissions and sustainability.

## Figures and Tables

**Table 1:** Main characteristics of each of the focus groups (before and after).

Focus Groups/ Attributes	Availability of Private Driver	Income	Family Structure	Age
**1. Set 1 (before)**				
1 Working (6 participants) × 2 sets	5 with private drivers 1 no private drivers	3 high income 3 medium income	3 with larger families including three generations 3 with smaller families including two generations	3 older 3 younger
2 Non-working (5 participants) × 2 sets	3 with private drivers 2 no private drivers	3 high income 2 medium income	3 with larger families including three generations 2 with smaller families including two generations	3 Older 2 younger
3 University students (4 participants) × 2 sets	1 with private drivers in the family 3 no private drivers	2 high income 2 medium income	2 with larger families including three generations 2 with smaller families including two generations	1 on campus 3 live with family
**2. Set 2 (after)**				
Focus groups/attributes	Availability of private car	income	Family structure	Age
4 Working (6 participants) × 2 sets	5 with private car 1 no private car	3 high income 3 medium income	3 with larger families including three generations 3 with smaller families including two generations	3 older 3 younger
5 Non-working (5 participants) × 2 sets	3 with private car 2 no private car	3 high income 2 medium income	3 with larger families including three generations 2 with smaller families including two generations	3 Older 2 younger
6 University students (4 participants) × 2 sets	1 with private car 3 no private car	2 high income 2 medium income	2 with larger families including three generations 2 with smaller families including two generations	1 on campus 3 live with family

## Data Availability

Not applicable.
